# Agar–Agar Gels Carrying Curative and Preventive Agents Against Helminths: An In Vitro Compatibility Evaluation

**DOI:** 10.3390/gels11070542

**Published:** 2025-07-12

**Authors:** Izaro Zubiría, Inês Abreu, David Boso, Gustavo Pérez, Cristiana Cazapal, Rita Sánchez-Andrade, María Sol Arias, Adolfo Paz-Silva, José Ángel Hernández, Mercedes Camiña

**Affiliations:** 1 Control of Parasites Group (COPAR, GI-2120), Department of Animal Pathology, Faculty of Veterinary, University of Santiago de Compostela, 27002 Lugo, Spain; izaro89@gmail.com (I.Z.); inesabreu.ramos@usc.es (I.A.); david.boso@rai.usc.es (D.B.); cristiana.cazapal@usc.es (C.C.); rita.sanchez-andrade@usc.es (R.S.-A.); mariasol.arias@usc.es (M.S.A.); adolfo.paz@usc.es (A.P.-S.); 2 Laboratory of Helminthology, National Centre for Disciplinary Research in Animal Health and Innocuity (CENID-SAI), National Institute for Research in Forestry, Agriculture and Livestock-Ministry of Agriculture and Rural Development, (INIFAP-SADER), Jiutepec 62550, Mexico; tavopzaz@gmail.com; 3 Department of Physiology, Faculty of Veterinary Medicine of Lugo, University of Santiago de Compostela, 27002 Lugo, Spain; merchi.camina@usc.es

**Keywords:** endoparasites, animals, deworming, *Mucor circinelloides*, prevention

## Abstract

The global market size of animal parasiticides was valued at USD 12.9 billion in 2024. Animal deworming only results in temporary cures with little to no preventive effects; therefore, a strategy that combines animal deworming with prevention is essential in improving the control of helminths. The effectiveness of co-administrating curative and preventive agents and their compatibility were considered based on the parasitophagous fungus *Mucor circinelloides*, which was developed in edible agar–agar (red seaweed)-carrying dewormers. Accordingly, Petri dishes were prepared with either a biopolymer alone (control, G-C) or with the anthelmintic piperazine (550, 1102, 2210, and 5500 mg/plate) or levamisole (37.5, 75, 150, and 300 mg/plate) and were used to culture the fungus *Mucor circinelloides*. Strong fungal growth and high numbers of spores were observed in the presence of the anthelmintics. No differences were measured between the control plates and those containing parasiticide drugs. Similar mycelial growth patterns and sporogenesis rates were recorded for different amounts of each anthelmintic. In conclusion, this novel formulation based on biopolymers containing anthelmintics and enriched with the parasitophagous fungus represents a highly promising tool to consider for jointly deworming animals and minimizing the risks of helminth infection. Further studies are in progress to confirm these in vitro results.

## 1. Introduction

The administration of therapeutic agents is very common among humans and animals through different routes; the most common methods are oral and parenteral administration, including intravenous, intramammary, intramuscular, and subcutaneous pathways [1,2]. For several decades, other diverse routes have been being explored, specifically to improve new pharmacological treatments among humans. In many cases, these have focused on the administration and release of drugs. In terms of veterinary care, major efforts are made to ensure easy and safe administration to animals and their keepers [3].

The precise control of animal parasites is a major and frequent topic of concern for veterinarians. The global market size of animal parasites (referring to the medical cost of deworming animals infected by parasites) was valued at USD 12.9 billion in 2024 [4]. The annual cost of treating livestock infected with helminths was estimated at EUR 1.8 billion in the EU, USD 7.11 billion in Brazil, more than USD 1.41 billion in Mexico, and USD 8.5 billion in the USA [5,6]. The control of parasites is a serious problem because many of them can be transmitted to humans; i.e., there are zoonoses caused by flatworms (*Fasciola hepatica*), tapeworms (*Echinococcus* spp., *Taenia* spp.), roundworms (*Toxocara canis*, *Ascaris suum*, or *Baylisascaris procyonis*), hookworms (*Ancylostoma* spp., *Uncinaria* spp.), and scabies mites. This remains an issue to be resolved, despite the many successful molecules that have been developed and marketed in the last century to target different groups of pathogens (Table 1).

The most frequently available drug formulations for the deworming of different species include liquids, pastes, granules, pills, and powders, which are administered orally to animals; however, their administration depends on the animal species and the management regime used to treat livestock (Table 1). Hence, special immobilization is sometimes required to ensure that animals receive the appropriate dose of the treatment assigned by a clinician, particularly in aggressive/angry pets or large livestock species [3].

The life-cycle of many helminths consists of two phases: in the internal phase, the immature stages are inside the animals (host) and develop to mature parasites, causing major damage [3,4,5]. The adult parasites shed eggs or larvae through their feces, which embed the infective stages into the soil (external phase). Obviously, these “free-living” stages remain unaffected by the antiparasitic strategies routinely applied to animal species; as a result, they can become reinfected quickly. Therefore, parasite control should not be based exclusively on deworming but integrated into preventive strategies to minimize the risk of infection among animals by reducing the presence and survival of parasites in the soil (environment) through land management actions to destroy their habitat [7]. Another possibility consists of rotational grazing to prevent these animals being exposed to elevated parasite burdens [8]. These strategies are very useful in avoiding additional early deworming, which could lead to the overuse and incorrect dosing of parasiticide drugs [7,8].

**Figure 1:**
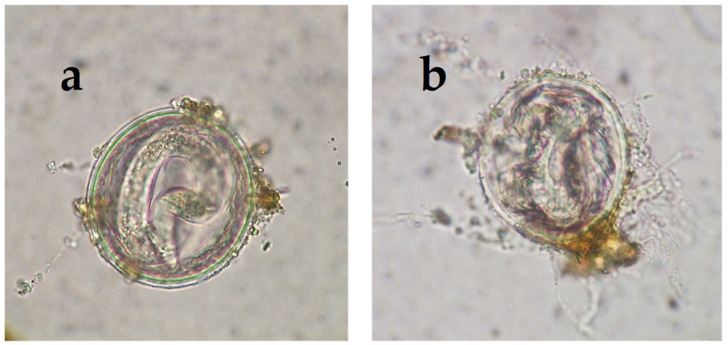
Some filamentous fungi, such as *Mucor circinelloides,* connect to the cuticle of certain helminth eggs (*Toxocara canis* in the picture) and penetrate inside, destroying the embryo. (**a**) Intact infective egg of the zoonotic roundworm *Toxocara canis*; (**b**) non-viable (uninfective) eggs of *T. canis* with the eggshell broken by *M. circinelloides* hyphae.

Some filamentous fungi that are normally present in the soil have demonstrated antagonism against various stages of helminths, including oocysts/cysts, eggs, and larvae. *Mucor circinelloides*, *Purpureocillium lilacinum*, *Trichoderma atrobrunneum*, and *Pochonia chlamydosporia* are saprophytic fungi. They are frequently found in soil and are involved in nutrient cycling and the decomposition of organic matter. These fungal species are also known as ovicides because they are able to develop a mycelium in the presence of eggs of helminths such as trematodes, cestodes, and nematodes that adheres to and penetrates the cuticle/eggshell. This eliminates the embryo and removes the inner content, breaking the eggshell that protects the internal structures [9] (Figure 1) and thus reducing the risk of infection.

Multiple industrial applications have been highlighted for *M. circinelloides*, besides its ability to destroy the oocysts of certain protozoan parasites and the eggs of numerous helminths [8,10]. These include its ability to produce a wide variety of hydrolytic enzymes, biodiesel, carotenoids, ethanol, and biomass with important nutritional value [11,12].

The most frequently used method of distributing parasitophagous fungi among animals is the oral administration of their spores through liquid solutions that have been mixed either with cereals prior to being ingested or nutritional pellets enriched with the fungi during their manufacturing [8,13,14]. Other novel formulations involve the ingestion of fresh or lyophilized gelatin enhanced with collagen, which has been successfully tested. It is an easy, safe, and helpful method that prevents infection by helminths among dogs and herbivores [15].

The usefulness of some marine sources and their easy acquisition have led to a growing interest in novel and practical applications [16]. Among these resources, marine biopolymers have been used for decades in the food and cosmetic industries and, more recently, for drug delivery in humans [16,17,18]. In the present investigation, the suitability of simultaneously taking curative and preventive action against helminths infecting animals was analyzed in vitro, using anthelmintics and parasitophagous fungi. For this purpose, the ability of spores of the fungus *M. circinelloides* (an antagonist of the oocysts/cysts/eggs of several parasites) to develop a mycelium that develops new spores (sporogenesis) in the presence of anthelmintics has been evaluated on an edible biopolymer (agar–agar extracted industrially from various types of red algae). Different concentrations of the anthelmintics piperazine and levamisole commonly administered to dogs, pigs, cattle, and horses were added.

## 2. Results and Discussion

### 2.1. The Development of M. circinelloides in Different Culture Media

**Figure 2:**
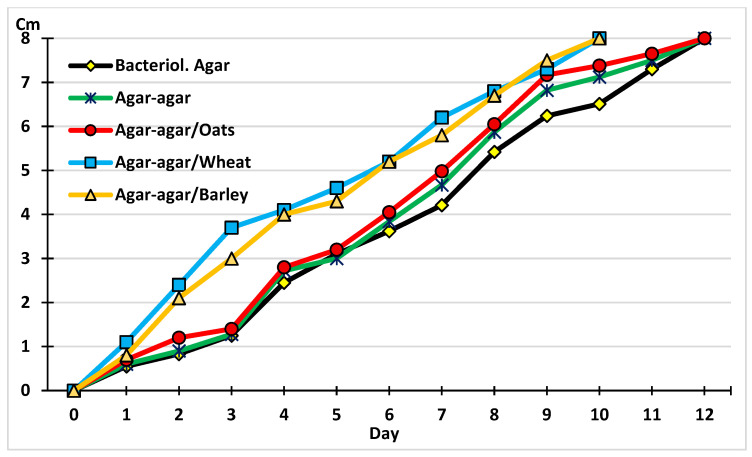
The growth of *M. circinelloides* mycelium in different media.

To favor the development (mycelial growth) of *M. circinelloides*, five culture media were prepared consisting of either a bacteriological agar, agar–agar, or an agar–agar with added oats, wheat, or barley (Figure 2). A notable linear growth was observed in all the media, with the best results produced by adding wheat or barley. These additions caused the fungus to fill the plates in ten days compared to twelve days in the other groups, though these differences were not significant (F = 1.131, *p* = 0.312). These results agree with previous investigations involving the addition of wheat to enhance the development (growth) of parasitophagous fungi such as *Duddingtonia flagrans*, *M, circinelloides*, *Pochonia chlamydosporia,* and *Clonostachys rosea* [10,19].

### 2.2. The Development of M. circinelloides in the Presence of Anthelmintics

**Figure 3:**
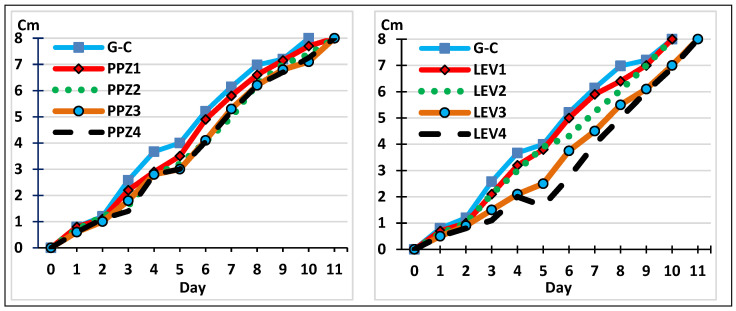
The growth of *M. circinelloides* mycelium in the presence of different quantities of piperazine (**left**) and levamisole (**right**). PPZ_1_: 55; PPZ_2_: 110; PPZ_3_: 221; PPZ_4_: 550 mg piperazine/plate. LEV_1_: 3.75; LEV_2_: 7.5; LEV_3_: 15; LEV_4_: 30 mg levamisole/plate. G-C: control without anthelmintics.

The next step involved studying the development of the fungus in the presence of two anthelmintics routinely administered to pigs, cattle, and horses. As shown in Figure 3, fungal growth was observed in the control plates (using agar–agar without anthelmintics) and with different concentrations of piperazine or levamisole added to the agar–agar plates. No differences were observed between the controls and the plates containing anthelmintics (F = 1.131, *p* = 0.312) or between the different concentrations of both piperazine or levamisole (F = 1.131, *p* = 0.312, and F = 1.382, *p* = 0.249, respectively).

Few investigations have evaluated the development of parasitophagous fungi exposed to therapeutic agents. Recent research has demonstrated that *M. circinelloides* is not affected by the presence of lasalocid or amprolium, which are active drugs against chicken protozoa, or albendazole, fenbendazole, levamisole, and ivermectin, which are antagonists of gastrointestinal helminths [10]. Analyzing the presence of benzimidazoles revealed that the in vitro growth of the ovicide fungi *Verticillium chlamydosporium* and *Purpureocillium lilacinum* was more sensitive to albendazole than triclabendazole [20]. It has also been found that *P. lilacinum* and the larvicide fungi *Arthrobotrys oligospora* and *Duddingtonia flagrans* are inhibited at certain concentrations of the anthelmintics albendazole, thiabendazole, ivermectin, levamisole, and closantel [21].

### 2.3. Sporogenesis of M. circinelloides in Different Media

**Figure 4:**
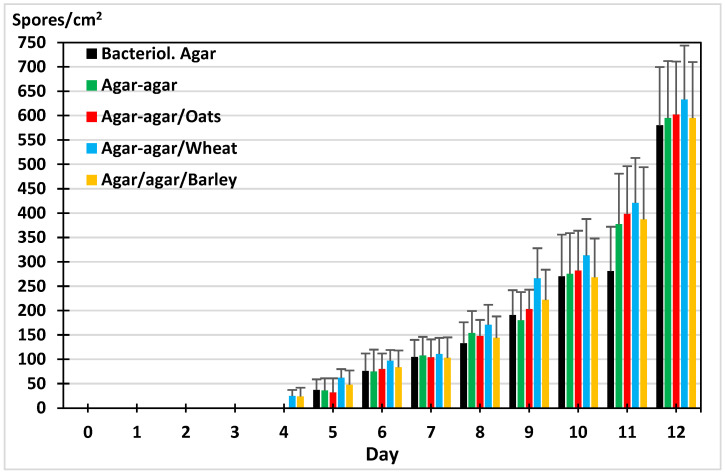
Sporogenesis of *M. circinelloides* in different culture media.

The production of chlamydospores was recorded after four days on agar–agar supplemented with wheat or barley and five days in media without cereals or with oats. A consistent increase was observed in the presence of wheat, but no differences were observed with respect to the media composition (F = 0.372, *p* = 0.829) (Figure 4).

It has been demonstrated that fungal sporogenesis is markedly enhanced by the addition of wheat to solid media composed of bacteriological agar [19,22]. The results show that high amounts of chlamydospores of *M. circinelloides* can be obtained in agar–agar cultures without supplementation with wheat, oats, or barley, which underlines the usefulness of this medium in developing protocols based on the utilization of this parasitophagous fungus. The effectiveness of co-administrating curative agents (piperazine, levamisole) and *M. circinelloides* and their compatibility were also verified in this research [Section 2.2 and Section 2.3].

### 2.4. Sporogenesis of M. circinelloides in the Presence of Anthelmintics

**Figure 5:**
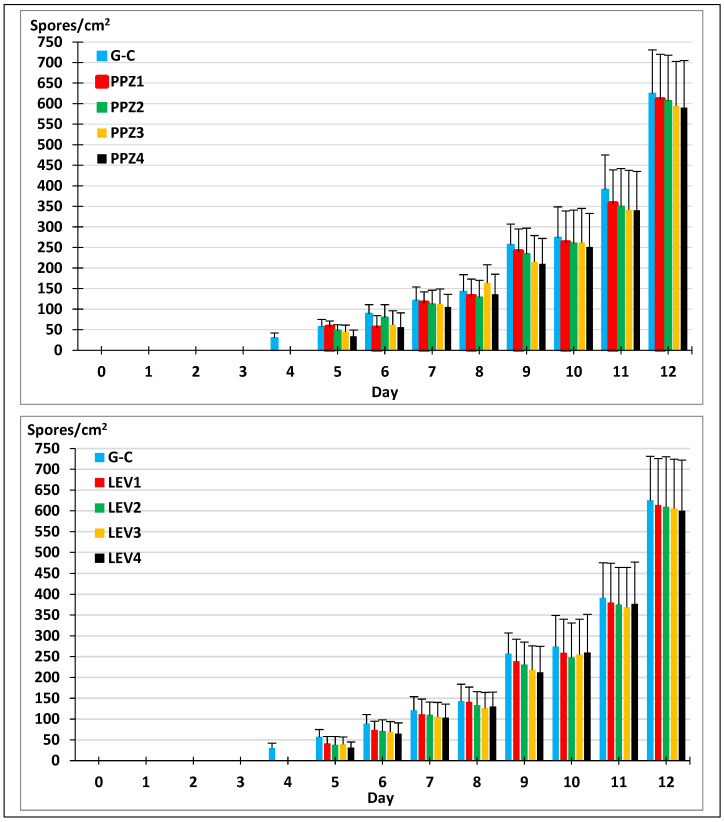
Sporogenesis of *M. circinelloides* in the presence of anthelmintics. PPZ_1_: 55; PPZ_2_: 110; PPZ_3_: 221; PPZ_4_: 550 mg piperazine/plate. G-C: control without anthelmintics. LEV_1_: 3.75; LEV_2_: 7.5; LEV_3_: 15; LEV_4_: 30 mg levamisole/plate.

The kinetics of the sporogenesis of *M. circinelloides* cultured in agar–agar with different concentrations of anthelmintics is drawn in Figure 5. Chlamydospores were recorded from the fourth day in media without anthelmintics (controls), and the levels increased significantly in all the plates until the end of the assay (day 12), with no differences regarding the concentration of piperazine (F = 0.148, *p* = 0.964) or levamisole (F = 0.110, *p* = 0.979).

These results emphasize the fact that the production of *M. circinelloides* is not influenced by the presence of therapeutic agents routinely administered to control helminths in dogs and herbivores. This confirms the idea that both anthelmintics and the ovicidal fungus can be administered simultaneously, providing a unique tool with the dual purpose of controlling parasites by acting on the animals (parasiticide) and on the environment (fungus).

### 2.5. The Analysis of Possible Applications

Helminths are a broad group of parasites that affect different organs in mammals, birds, and humans. Many of these pathogens are endoparasites, such as trematodes, cestodes, and nematodes (strongyles, ascarids, or roundworms, and trichurids or whipworms), capable of damaging the digestive tract and causing important clinical problems characterized by diarrhea, anorexia, anemia, weight loss, reduced productivity, infertility, and even death [23]. This supports the need for curative actions in infected animals, for which many successful anthelmintics are commercially available. As mentioned previously, deworming acts as a cure for infected individuals; however, these species can quickly reinfect, requiring additional deworming applications [24]. The misuse of this treatment (especially abuse or underdosing) has been associated with the emergence of anthelmintic resistance, i.e., strains of parasites that survive the administration of certain parasiticides and are also able to transmit this ability (hereditary) [25].

Despite its capability for success, deworming is insufficient to control parasites as individuals experience rapid reinfection. As such, preventive measures are essential in minimizing the risk of infection and, consequently, the need for frequent deworming. A promising option involves the use of parasitophagous fungi, which are mainly distributed as spores that develop into hyphae (mycelium) and are active against some parasitic stages in the soil, with the ability to generate new spores [26,27].

**Figure 6:**
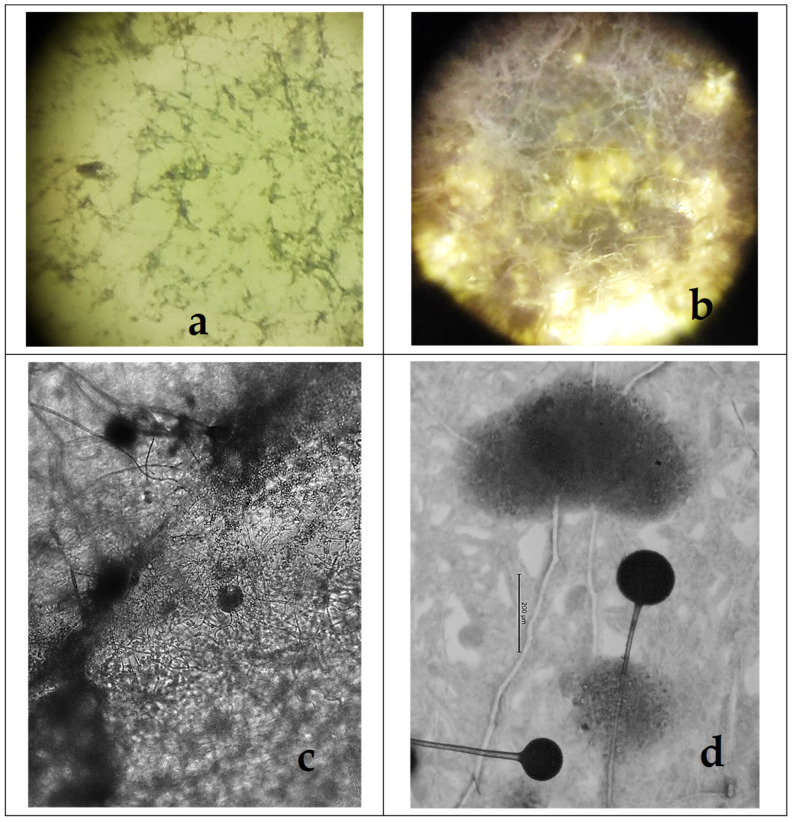
The parasitophagous fungus *M. circinelloides* developed properly in the mycelium in the presence of different concentrations of piperazine and levamisole. (**a**) Mycelium in plates with piperazine (5×); (**b**) levamisole (10×). (**c**) Sporogenesis and (**d**) mature sporangia showing sporogenesis (40×).

Based on available curative and preventive actions, a significant and logical question is whether the combined use of therapeutic agents and fungi is viable [21,28]. Data collected during this research revealed that *M. circinelloides* does not exhibit any defects when cultured with piperazine or levamisole, and a marked development was recorded in agar–agar plates (Figure 6). Moreover, it has been demonstrated that this fungus can adequately develop when cultured with the correct dose of piperazine corresponding to dogs weighing between 2.5 and 27 kg, or to pigs, cattle, and horses between 5 and 50 kg (Table 2). Similarly, no adverse effects have been identified when *M. circinelloides* is used in the presence of the recommended dosage of levamisole for 3.7–30 kg dogs, or pigs, cattle, and horses between 5 and 50 kg.

In the current investigation, it is demonstrated for the first time that the growth and sporogenesis of *M. circinelloides* are not influenced by the presence of piperazine and levamisole at different concentrations. These results have been observed in agar–agar with or without cereals; this new formulation is helpful in designing more strategies to control parasites among animals. Prior investigations conducted in collagen gelatins enriched with the spores of parasitophagous fungi demonstrated that this formulation provides reliable and promising results concerning the practical possibility of controlling certain parasites. It offers an easy tool through which spores can reach the feces of animals and act against the pathogens in that environment [15]. This antagonistic effect is responsible for minimizing the parasitic burden in the soil and, accordingly, the risk of infection among animals. The ultimate benefit of this would be the proper and less frequent administration of deworming practices, with positive consequences for the environment and animal health. This raises a very interesting question based on the practical use of agar–agar containing a parasiticide plus a parasitophagous fungus. It should be administered to animals infected by parasites and especially to those kept in small–medium plots on farms or zoos or even among pets living in landscaped areas. It would also be very useful in the case of animals that are not very docile or to avoid causing stress to them.

Data recorded in the current investigation indicate that *M. circinelloides* can be used simultaneously with the anthelmintics piperazine and levamisole. Moreover, considering the unique research carried out involving this fungus [10], the spectrum of anthelmintics has been significantly improved, including some antiprotozoal drugs. This seems highly relevant because it provides more security when designing strategies that simultaneously focus on deworming and preventive measures. It is important to note that it would be ineffective to stop the administration of parasitophagous fungi for a long period of time because the deworming treatment could affect them.

Due to their gelling properties, collagen-based gelatins have been successfully used for the distribution of spores of *M. circinelloides* and *D. flagrans* as fresh or dried parcels [15]. The availability of edible solutions offers a new and viable tool for the administration of therapeutic agents that can be taken voluntarily, enhancing complete ingestion and guaranteeing the expected effect. As stated previously, deworming constitutes the main (and in most cases the only) measure to control parasites of veterinary importance; preventive actions are seldom considered, mostly due to the absence of options. Despite this, the administration of therapies in some animal species may be problematic due to the difficulty in administering the necessary dose. Many oral formulations involve unintentional or unwanted ingestion, so some individuals need to be immobilized and/or relaxed; the same occurs when injections are applied. Some preparations entail the repeated ingestion of large volumes, resulting in problems when deworming undocile pets or even large animals such as horses. These issues could be easily solved if agar–agar-based products containing anthelmintics were available.

Another interesting issue lies in the fact that deworming focuses on the elimination/expulsion of adult parasites living inside the animals (their hosts). Even after the successful administration of parasiticidal drugs, the eggs of these pathogens are only minimally affected at most. Animals are only temporarily free of parasites because their eggs continue to evolve to infective stages, creating an ongoing risk of reinfection. This study’s results demonstrate that the control of parasites in animals could be significantly improved if agar–agar-based products containing anthelmintics were enriched with spores of an ovicide fungus such as *M. circinelloides*, which has beneficial action against parasites present in the soil [8,9,14,15].

To the best of our knowledge, the current research constitutes the first analysis of the possibilities of agar–agar as a biopolymer for the simultaneous delivery of parasiticide drugs and chlamydospores of the parasitophagous fungus *M. circinelloides*. This investigation is essential in creating novel strategies to control helminth infections in animals, including effective deworming and reducing reinfection risks. Previous investigations have involved other biopolymers for controlled drug delivery, such as chitosan, dextran, or polyesters [2,29]. It is important that collagen-based formulations be successfully administered for the distribution of different parasitophagous fungi spores in animals. These beneficial organisms are active against parasitic stages in the soil and develop an antagonistic role against helminths [8,15].

## 3. Conclusions

Edible agar–agar provides a medium gel for the parasitophagous fungus *M. circinelloides*, which can develop properly and produce high amounts of spores; these biological properties are not affected by the presence of piperazine or levamisole; these are anthelmintics routinely administered to pets and farm animals. Accordingly, agar–agar biopolymers represent promising strategies for the control of helminths, with the aim of curing animals (anthelmintics) and preventing new infections by minimizing soil contamination (parasitophagous fungus). New investigations are being carried out into the rheological characterization of this novel formulation, including testing among pets and livestock.

## 4. Materials and Methods

The *Mucor circinelloides* strain CECT 20,824 was utilized in the current investigation and isolated by the COPAR Research Group (GI-2120; USC) from livestock feces and farm soil samples; it was then deposited at the Spanish Type Culture Collection (CECT, Valencia, Spain) [22].

### 4.1. Experimental Design

The design of the current investigation comprised evaluating a proper medium containing agar–agar for culturing the fungus *M. circinelloides*. With this aim, various media were first developed and then analyzed for growth and sporogenesis, i.e., for the production of chlamydospores. These are resting and thick-walled spores produced by different fungi for their dissemination. They were widely administered to different animal species with the aim of ensuring their presence in the soil, where these organisms can develop and destroy the infective stages of helminths [13,14,19]. Subsequently, we evaluated the ability of *M. circinelloides* spores to develop a mycelium that, in turn, developed new spores in the presence of anthelmintics.

#### 4.1.1. The Development of *M. circinelloides* in Agar–Agar

To check the development of parasitophagous fungus in edible agar–agar, 5 sets of 6 Petri plates (8 cm diameter) were prepared by adding (per L distilled water) (a) 20 g of bacteriological agar (Sigma-Aldrich, Madrid, Spain); (b) 10 g of edible agar–agar (extracted from various types of red seaweed, *Rhodophyceae*; Portomuiños, A Coruña, Spain); (c) 10 g of agar–agar and 25 g of wheat flour; and (d) 10 g of agar–agar and 25 g of oat flour.

All the prepared media were sterilized in an autoclave (121 °C, 15 min), and once cooled, they were poured into Petri plates under a laminar flow hood. Finally, single square agar blocks (7 × 7 mm) were cut from actively growing *M. circinelloides* stock cultures using a microspoon spatula (7 mm width); these were used to inoculate the plates (Figure 6), which were maintained at RT (16–22 °C) for 8–12 days (until the plate was filled). This scheme was performed in duplicate. Only one agar block was placed on one side of each plate. This assay was conducted in duplicate.

#### 4.1.2. The Development of *M. circinelloides* in Agar–Agar with Added Anthelmintics

In total, 3 groups of 10 plates each were prepared by mixing distilled water, edible agar–agar, and wheat flour, as mentioned previously. As summarized in Table 3, the group G-PPZ had different concentrations of the anthelmintic piperazine citrate added (Piperacina Syva^®^ 1000 mg/g, Laboratorios SYVA S.A., Madrid, Spain); the G-LEV received another anthelmintic, levamisole hydrochloride (Panvermin Oral^®^ 150 mg/g, Laboratorios SYVA S.A.U., Madrid, Spain), and the control group (G-C) remained without anthelmintics. As explained in Table 2, the quantities of piperazine added to the plates corresponded to the dosage indicated for the deworming of dogs between 2.5 and 27 kg, as well as pigs, cattle, and horses between 5 and 50 kg. Likewise, the quantities of levamisole matched the recommended dosage for dogs between 3.7 and 30 kg, as well as pigs, cattle, and horses between 5 and 50 kg.

As described above, the plates were placed in a single agar block (7 × 7 mm) at one side and then kept at RT (16–22 °C) until the plate was covered using mycelia developed by the fungus *M. circinelloides* (8–12 days). This scheme was performed in duplicate.

#### 4.1.3. Evaluating the Development of *M. circinelloides*

The possible influence of the anthelmintics piperazine and levamisole on the survival and development of *M. circinelloides* was assessed by measuring the growth of the mycelium and the number of chlamydospores produced. All the plates were examined daily under a loupe at 5, 10, or 20×, and the progression (growth) was drawn using a marking pen; the results are expressed in cm [22] (Figure 6).

**Figure 7:**
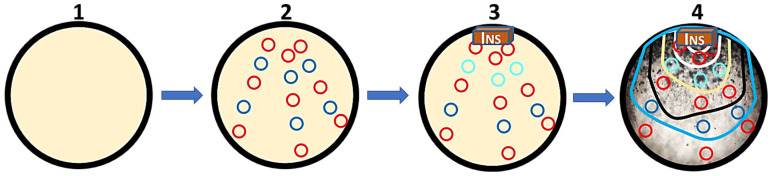
Petri plates were prepared with edible gelatin with or without anthelmintics. (**1**) Then, fifteen 1 cm^2^ circles were drawn on the back side to improve the chlamydospore count; (**2**) a plug of fungal inoculum (INS) was placed on each plate near an edge on one side; and (**3**) mycelial development was recorded daily with a marker pen (**4**).

The number of chlamydospores that appeared daily was also recorded using a light microscope at 40×. For this purpose, at the beginning of the assay, a total of 15 1 cm^2^ circles were drawn on the back side of the plates (Figure 7) and examined; the counts were expressed as the number of chlamydospores per cm^2^.

#### 4.1.4. Data Analysis

The data obtained from the current investigation were first analyzed using the Levene and Kolmogorov–Smirnov tests to check whether they followed a normal distribution. As a result of this observation, the data collected were evaluated via analysis of variance (ANOVA) at a significance level of *p* < 0.05.

All tests were performed with the statistical package IBM SPSS Statistics, version 28 (SPSS Inc., Chicago, IL, USA).

## Figures and Tables

**Table 1:** Several anthelmintics can be administered using different routes to control helminths in animals.

Active Principle	Formulation	Target
Piperazine	Pills, powder	Roundworms
		Pinworms
		Hookworms
Levamisole	Liquid, powder	Trichostrongylids
		Hookworms
		Roundworms
Albendazole	Liquid, paste	Trematodes
		Trichostrongylids
		Hookworms
		Roundworms
Ivermectin	Liquid, paste, injectable, poured on	Trichostrongylids
		Hookworms
		Roundworms
		Whipworms
		Ectoparasites

**Table 2:** The parasitophagous fungus *M. circinelloides* can grow and develop properly in the presence of different dosages of helminths corresponding to dogs, pigs, cattle, and horses.

Group	Anthelmintic	Quantity Added/Plate (mg)	Corresponding Dosage	
			Dogs (kg)	Pigs, Cattle, and Horses (kg)
PPZ_1_	Piperazine	546	2.5	5
PPZ_2_		1102.5	5.5	10
PPZ_3_		2205	11	20
PPZ_4_		5495	27	50
LEV_1_	Levamisole	37.5	3.7	5
LEV_2_		75	7.5	10
LEV_3_		150	15	20
LEV_4_		375	30	50

**Table 3:** The development of *M. circinelloides* was analyzed in Petri plates prepared with edible gelatin and added anthelmintics, except for the control group (G-C).

Group	Name	Piperacina Syva^®^ (g Added/Plate)	Piperazine (mg/Plate) *^1^	Panvermin^®^ (g Added/Plate)	Levamisole (mg/Plate) *^2^
G-C	G-C	-	-	-	-
G-PPZ	PPZ_1_	1.56	546		
	PPZ_2_	3.15	1102.5		
	PPZ_3_	6.3	2205		
	PPZ_4_	15.7	5495		
G-LEV	LEV_1_			0.25	37.5
	LEV_2_			0.5	75
	LEV_3_			1	150
	LEV_4_			2.5	375

*^1^ Recommended dosage for dogs, 200 mg/kg of body weight; recommended dosage for pigs, cattle, and horses, 110 mg/Kg b.w. *^2^ Recommended dosage for dogs, 10 mg/kg b.w.; recommended dosage for pigs, cattle, and horses, 7.5 mg/Kg body weight.

## Data Availability

The raw data supporting the conclusions of this article will be made available by the authors upon request.
